# Treatment outcomes of surgery followed by short-course every other day radiotherapy in keloid

**DOI:** 10.1186/s13014-024-02488-5

**Published:** 2024-07-17

**Authors:** Wei Zhou, Bing Li, Yutian Yin, Lihua Zhang, Yan Zhou, Lin Xu, Jian Zang, Lina Zhao

**Affiliations:** 1 Department of Radiation Oncology, Xijing Hospital, Fourth Military Medical University, 15 West Changle Road, Xi’an, China; 2 State Key Laboratory of Holistic Integrative Management of Gastrointestinal Cancers, Department of Radiation Oncology, Xijing Hospital, Fourth Military Medical University, Xi’an, China; 3 Dermatology Department, Xijing Hospital, Fourth Military Medical University, Xi’an, China

**Keywords:** Keloid, Postoperative radiotherapy, Electron-beam, Local control

## Abstract

**Background:**

Postoperative radiotherapy can significantly reduce keloid recurrence. However, consensus on the optimal radiotherapy dose and treatment schedule remains elusive. This study aims to evaluate the effectiveness of surgery followed by a short-course of radiotherapy administered every other day for keloid treatment.

**Materials/Methods:**

We conducted a retrospective analysis of 498 patients with keloids treated at our institution between January 2010 and December 2017. All patients underwent electron beam irradiation at a dose of 16 Gy, delivered in four fractions every other day, starting within 24 h post-surgery. The primary endpoint of the study was the local control rate.

**Results:**

A total of 130 (26.5%) keloids recurred after a median follow-up of 68.1months (42.6-129.9 months). The local control rates at 1 year, 3 years and 5 years for all patients were 89.5%, 82.5% and 81%, respectively. The highest recurrence rate was observed in keloids located in the chest region (50.8%), followed by the suprapubic (47.8%), head and neck (38.8%), limbs (33.3%) and ear (14%). Both multivariate and univariate analyses identified the presence of pain and or pruritus as an independently prognostic factor for keloid recurrence (*p*<0.0001). The local control rates at 1-year, 3-years and 5-years for patients with or without symptom of pain or pruritus were 45% vs. 98.8%, 12.5% vs. 95.9%, and 8.8% vs. 95%, respectively (HR:37.829, 95%CI: 24.385–58.686, *p*<0.001). In the ear keloid subgroup, the 1-year, 3-year and 5-year local control rates for patients with pruritus were significantly lower than those without pain or pruritus (60.0% vs. 97.9%, 26.7% vs. 94.7%, 26.7% vs. 94.3%, HR:30.209, 95% CI:14.793–61.69, *p*<0.001). The same results were found in other location(*p*<0.001). During treatment and follow-up, two patients experienced infections, and one patient developed a cutaneous fibroblastoma.

**Conclusion:**

This study suggests that a combination of surgery followed by short-course, every-other-day radiotherapy can yield satisfactory local control rates for keloids. Pain and or pruritus symptom was an independently prognostic factors for recurrence of keloid. To further validate these results, a prospective randomized controlled trial is recommended.

**Supplementary Information:**

The online version contains supplementary material available at 10.1186/s13014-024-02488-5.

## Introduction

Keloids are pathological scar tissues that develop as a result of skin trauma or spontaneous formation and subsequent overgrowth. They typically occur due to burns, piercings, tattoos, surgery, or other forms of skin trauma. Keloid formation is characterized by an imbalance in collagen synthesis and extracellular matrix production, coupled with reduced degradation of these components. Inflammatory mediators are believed to play a role in influencing collagen synthesis and remodeling of the extracellular matrix during scar healing. Overactivation of keloid fibroblasts, driven by the overexpression of inflammatory mediators, is associated with increased collagen synthesis and extracellular matrix production [1]. Keloids are characterized by their raised, irregularly shaped, and invasive growth beyond the boundaries of the original wound or skin injury. They do not resolve spontaneously and often present with symptoms such as pain or pruritus. People with darker skin such as blacks and Asians individuals are more likely to develop keloids, with an incidence of about 5–16% [2]. The increasing incidence and recurrence of keloid can seriously impact patient’s physical and mental health [3].

Keloid can be treated with corticosteroid injection, compression therapy, surgery, physical therapy, radiation therapy and other methods [4]. Corticosteroid injections are most commonly used treatment, with recurrence rates ranging from 5 to 50%. However, injections are often painful for most patients [5, 6]. Compression therapy is an effective treatment for keloid, but patient compliance is often poor because it always takes at least half a year [7]. Surgery alone has a high recurrence rate ranging from 45 to 100%, and published data indicate that using surgery as a single treatment for keloids is insufficient [8, 9]. Radiotherapy is often conducted after surgical resection because it can effectively prevent keloid recurrence by inhibiting angiogenesis and inflammatory response [10, 11]. A recent meta-analysis, which included 72 studies, reported that surgery followed by radiotherapy could achieved a lower recurrences of 22%, indicating the potential effectiveness of this treatment modality for keloid [12]. Dose-effect studies suggest that a biological effective dose (BED) of 30 Gy is considered the optimal radiation dose. Higher BED doses do not further reduce the recurrence rate but increase side effects [10, 13, 14]. However, there is still a lack of consensus on optimal postoperative radiotherapy dose and fractions for keloid, especially based on different keloid regions and patient main complaints.

The aim of this study was to investigate the efficacy of surgery followed by a short-course radiotherapy administered every other day, a common protocol in our institute for treating keloids. Additionally, the study aimed to analyze prognostic factors associated with keloid recurrence.

## Materials and methods

### Patients

We conducted a retrospective review of clinical data from keloid patients who received postoperative radiotherapy at Xijing Hospital from January 2010 to December 2017. We collected the following patient characteristics for analysis: gender, age, cause, longest axis, keloid location, number of keloids, treatment history before radiotherapy, and accompanying symptoms such as pain, pruritus, and duration of symptoms. All patients provided consent for the treatment protocol, and the study was conducted in accordance with the principles of the Declaration of Helsinki.

### Radiotherapy

Electron beam with energy of 4 MeV was used for radiotherapy. The target area of irradiation was 0.5–1 cm around the incision suture line, and the bolus with 0.5 cm was covered to increase the epidermal dose. At the same time, lead blocks were used to protect normal tissue. The total dose was 16 Gy with 4 fractions, administered once every other day. All patients received radiotherapy within 24 h after surgery.

### Acute and late toxicities

Radiotherapy-related toxicity was evaluated according to the Acute and Late Radiation Morbidity Scoring Criteria of Radiation Therapy Oncology Group (RTOG). The toxicities were evaluated after radiotherapy every 3 months for the first year and every 3–6 months for the next two years.

### Statistical analysis

The follow-up period was measured from the beginning of radiotherapy to the time of recurrence or the last follow-up before analysis. The primary endpoint was local control which was defined as any clinical evidence of a keloid developing at the incision site, regardless of the size, as determined with a focused physical examination by the treating radiation oncologist, dermatologist, plastic surgeon, or otolaryngologist in follow-up. Receiver operating characteristic (ROC) curves were used to convert the continuous variables into two subgroups at their cutoff values identified by recurrence. The Chi-square test was used to compare the categorical variables (expressed in frequency or percentage). The Kaplan–Meier curve was used to calculate estimated rates of the percent of local control. The differences in the time-to-event outcomes between groups were compared by log-rank tests. Univariate and multivariate Cox analyses were used to detect prognostic factors for local control. A two-tailed *P* value < 0.05 was considered as statistically significant difference. The SPSS (version 24.0, IBM, USA) was used for statistical analyses.

## Results

### Patient characteristics

Among the patients, there were 126 (25.3%) male and 372 female (74.7%). The median age was 25 years (range: 4–77 years). Of all patients, 32 (6.4%) had more than 2 keloids and 466 (93.4%) had a single keloid. The causes of keloids were 298(59.8%) piercing, 44(8.8%) scar, 63(12.7%) acne, 56(11.2%) trauma, and 37(7.4%) other respectively. The longest axis of keloids was less than 5 cm in 64.7% of cases, between 5 cm and less than 10 cm in 14.9% of cases, and greater than 10 cm in 20.5% of cases, respectively. Eighty-one patients (16.3%) experienced pain or pruritus as a symptom. Among these, 9 patients had pruritus for less than 6 months, 4 patients for 6 to 12 months, and 68 patients for more than 12 months. All patients received comprehensive surgical-based treatment before undergoing radiotherapy. This included 337 patients who had surgery alone, 18 who received surgery combined with compress therapy, 26 who had surgery combined with corticosteroid therapy, 82 who underwent surgery in addition to silicone treatment, and 35 who received more than three kinds of comprehensive treatments (Table 1). For patients who received surgery combined with other treatments, all previous treatments were administered before the new surgery plus radiotherapy, as these treatments were found to be ineffective.

**Table 1:** Baseline characteristics for patients

Variable	*N* (%)
Median Age (range)	25(range :4–77)
Sex	
male	126(25.3)
female	372(74.7)
Causes of keloids	
piercing	298(59.8)
scar	44(8.8)
acne	63(12.7)
trauma	56(11.2)
other	37(7.4)
Longest axis	
<5 cm	322(64.7)
≥ 5 cm,<10 cm	74(14.9)
≥ 10 cm	102(20.5)
Number of keloids	
1	466(93.6)
≥ 2	32(6.4)
Location	
ear	298(59.9)
head neck	44(8.8)
chest	63(12.7)
abdomen	32(6.4)
perineum	37(7.4)
limb and back	24(4.8)
Pain and or pruritus	
yes	81(16.3)
no	417(83.7)
Duration of pain or pruritus	
<6 month	9(11.1)
6-12month	4(4.9)
≥ 12month	68(84)
Treatment before radiotherapy	
surgery alone	337(67.7)
compression + surgery	18(3.6)
corticosteroid + surgery	26(5.2)
silicone + surgery	82(16.5)
compression + corticosteroid + silicone + surgery	35(7.0)

### Local control and prognostic factors

**Fig. 1:**
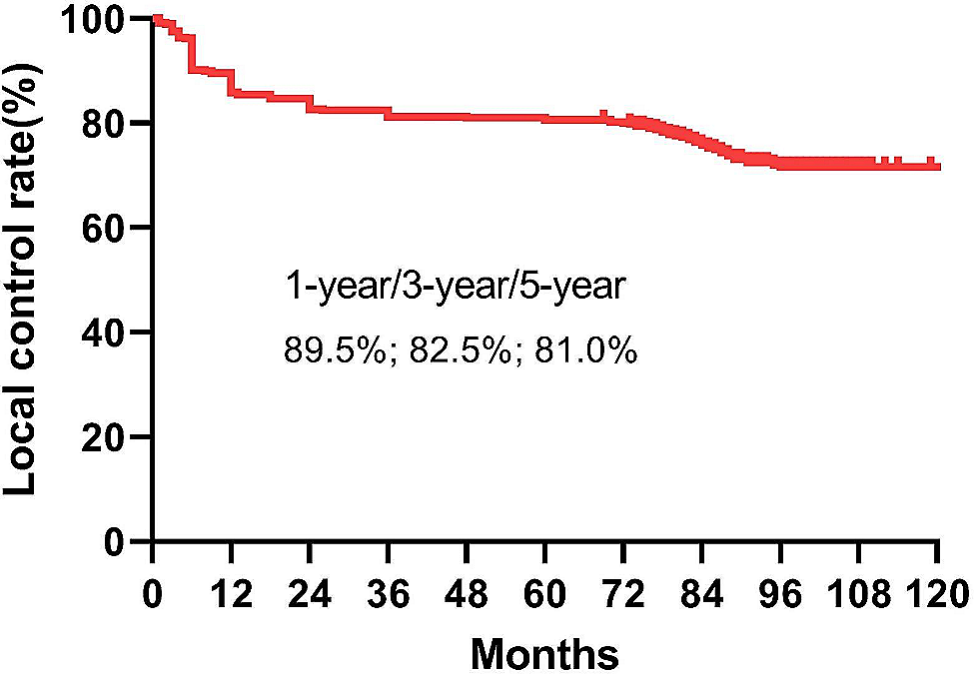
Kaplan-Meier analysis of local control rate for all patients

**Fig. 2:**
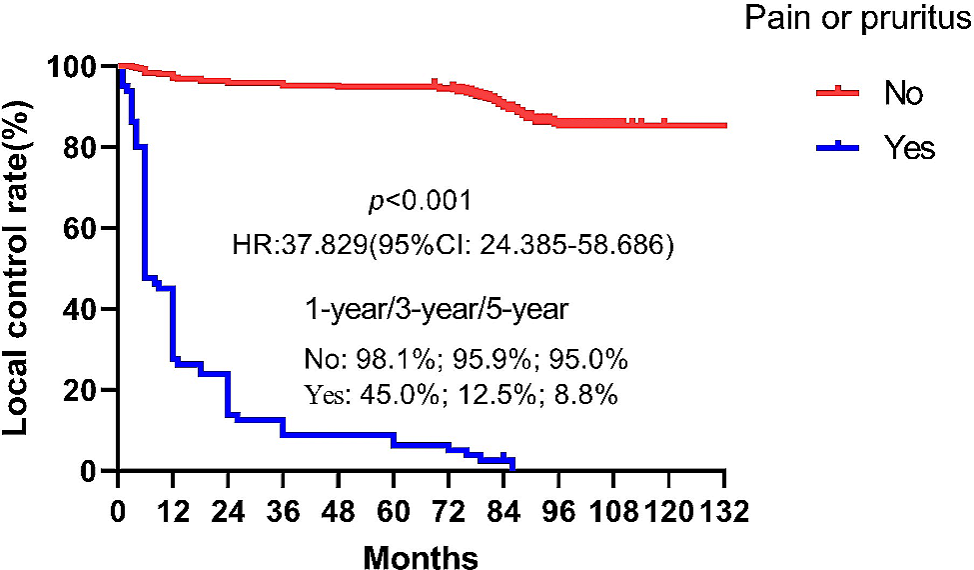
Kaplan-Meier analysis of local control rate for patients accompanied by pain and/or pruritus

At the last follow-up, 130 (26.5%) keloids had recurred after a median follow-up of 68.1 months (range: 42.6-129.9 months). In the group that underwent surgery only followed by radiotherapy, 65 recurrences were observed. Meanwhile, in the group receiving combined treatments with surgery followed by radiotherapy, 65 recurrences were found. The local control rates at 1 year, 3 years and 5 years for all patients were 89.5%, 82.5% and 81%, respectively (Fig. 1). Keloids in chest region had the highest recurrence rate (50.8%) (Supplemental Fig. 1), followed by suprapubic (47.8%), head neck (38.8%), limbs (33.3%) and the lowest recurrence rate of ear (14%) (Supplemental Fig. 2). Multivariate analysis was conducted to identify independent prognostic factors using the following covariates: age, sex, cause, longest axis, number of keloids, location, pruritus, duration of pruritus, treatment before radiotherapy. Pain and or pruritus was an independent prognostic factors for keloid recurrence detected by multivariate analysis (HR:14.926, 95%CI:6.760-32.958, *p*<0.0001)(Table 2). The local control rates at 1-year, 3-years and 5-years in patients with or without pain and or pruritus were 45% vs. 98.8%, 12.5% vs. 95.9%, and 8.8% vs. 95%, respectively (HR:37.829, 95%CI:24.385–58.686, *p*<0.001) (Fig. 2).

**Fig. 3:**
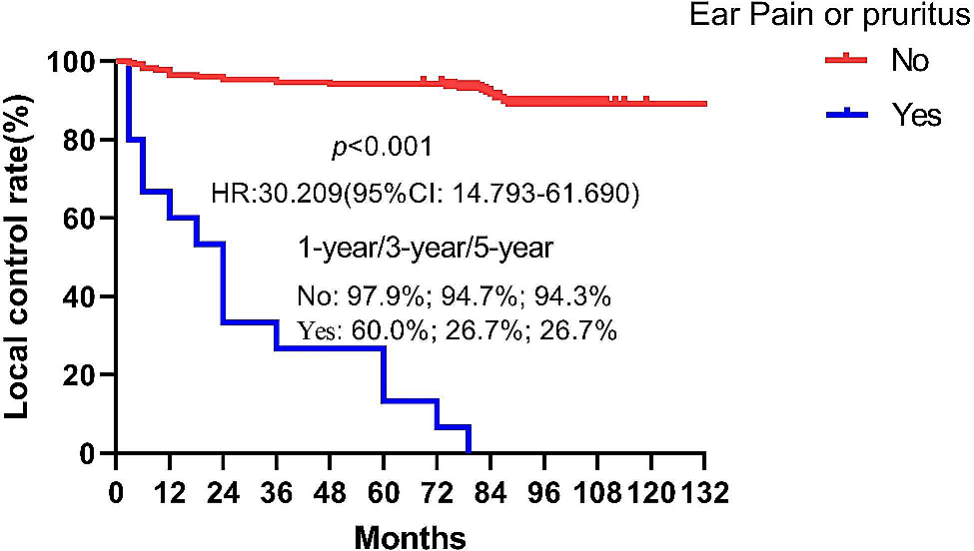
Kaplan-Meier analysis of local control rate of patients with ear keloid accompanied by pain and/or pruritus

The 1-year, 3-year and 5-year local control rates of patients with pain and or pruritus were lower than those without symptom in ear keloid subgroup (60.0% vs. 97.9%, 26.7% vs. 94.7%, 26.7% vs. 94.3%, HR:30.209, 95%CI:14.793–61.69, *p*<0.001)(Fig. 3). The same results were found in other location (Supplemental Table 1).

**Table 2:** Univariable and multivariate Cox analysis of local control

Variable	Univariable Cox analysis			Multivariate Cox analysis	
	HR (95%CI)	*p*		HR (95%CI)	*p*
Age					
<35	1.000				
≥ 35	2.059(1.422–2.983)	< 0.001			
Sex					
male	1.000				
female	0.460(0.323–0.654)	< 0.001			
Causes of keloids					
piercing	1.000				
scar	3.442(1.980–5.983)	< 0.001			
acne	4.906(3.094–7.779)	< 0.001			
trauma	3.137(1.857-5.300)	< 0.001			
other	3.685(2.097–6.475)	< 0.001			
Longest axis					
<5 cm	1.000				
≥ 5 cm,<10 cm	3.021(1.926–4.739)	< 0.001			
≥ 10 cm	3.539(2.389–5.242)	< 0.001			
Number of keloids					
1	1.000				
≥ 2	1.250(0.656–2.383)	0.498			
Location					
ear	1.000				
head neck	3.443(1.981–5.985)	< 0.001			
chest	4.908(3.095–7.782)	< 0.001			
abdomen	3.830(2.090–7.019)	< 0.001			
perineum	3.686(2.098–6.476)	< 0.001			
limb and back	2.304(1.035–5.130)	0.041			
Pain and/or pruritus					
no	1.000			1.000	
yes	37.829(24.385–58.686)	< 0.001		14.926(6.760-32.958)	< 0.001
Duration of pain or pruritus					
<6 month	1.000				
6-12month	2.029(0.338–12.167)	0.439			
≥ 12month	3.647(1.130-11.766)	0.030			
Treatment before RT					
surgery alone	1.000				
compression + surgery	1.582(0.637–3.930)	0.323			
corticosteroid + surgery	6.383(3.850-10.582)	< 0.001			
silicone + surgery	1.501(0.939–2.397)	0.089			
compression + corticosteroid + silicone + surgery	2.924(1.691–5.057)	< 0.001			

HR: Hazard ratios. CI: confidence interval. RT: radiotherapy

### Toxicities

During the radiation course and follow-up period, two patient experienced wound infection, and there were no cases of grade ≥ 2 radiation dermatitis, bleeding, or poor wound healing. During follow-up, one patient developed fibroblastoma in the radiation field at the suprapubic site. (Table 3).

**Table 3:** Acute and late toxicity

Toxicities	*N*
Acute adverse events	
dermatitis (≥ 2grade)	0
infection	2
bleeding	0
poor healing	0
Late adverse events	
fibroblastoma	1

## Discussion

It is widely accepted that surgical resection followed by radiotherapy is a rapid and effective method for treating keloids [11, 15, 16]. Many studies confirmed that surgery followed by radiotherapy could effectively reduce the recurrence rate of keloid and improve the quality of life of patients [9, 14, 17, 18]. However, in the past few years, there was no consensus on the dose and fraction of radiotherapy, and the treatment model varies from different institutes.

Studies have shown that postoperative radiation can significantly reduce the risk of keloid recurrence [7, 17, 19, 20]. A study included 124 patients with 250 keloid lesions reported that surgery followed by radiotherapy with 20 Gy in 5 fractions yielded an excellent local control rate of 94.4% [14]. Another study further verified the effectiveness of the radiotherapy regimen in keloid treatment, with a satisfactory local control rate of 84.8% [20]. Rei et al. reported adjusted radiotherapy regimens according to keloid locations achieved local control rate of 90%, such as 18 Gy radiation dose in 3 fractions to anterior chest wall and scapular region, 8 Gy radiation dose in 1 fraction to earlobes and 15 Gy in 2 fractions to other body site [10]. In this study, we reported comparable cumulative local control rates after a median follow-up of 68.1months. The local control rates of 1 year, 3 years and 5 years were 88.5%, 82.5% and 71%, respectively. This result was consistent with published data [14, 21]. Moreover, the correlation between local control and keloid location was established by our study. The keloid located on chest had the highest recurrence rate, while keloid located on ear had lowest recurrence rate. Similar results were also reported by other study [22]. The prescription BED was similar to other studies, and also achieved a satisfactory effect. But for high recurrence areas, such as the chest and abdomen, perineum, limbs and other high-tension areas, the prescribed BED in this study is slightly lower than some other studies, so this should be one of the reasons for the slightly higher recurrence rates in those areas in this study. It is still unclear why the lesion site can impact the recurrence of keloids. This may be explained by two reasons: ①the skin located on chest always has higher tension which is known to be a risk factor for keloid formation [23, 24]; ②the percentage of collagen, which is higher at chest and back, may contribute to keloid formation [25]. Therefore, the dose and fraction of radiotherapy should be delivered according to the keloid site.

In this study, we firstly reported that symptom of pain and or pruritus were independently prognostic factors for keloid recurrence regardless of keloid site. Although the exact reasons are not clear, these symptoms may be attributed to angiogenesis and fibroblast proliferation [26]. Compared keloid without pruritus symptom, keloid with pruritus always have increased number and density of dermal mast cells and their stored granules, which were considered as important factor to stimulate fibroblast activity and collagen formation [27]. Moreover, patients with high concentrations of inflammatory cells infiltration in keloid tissue were more susceptible to have pruritus symptom [28]. The factors mentioned above are closely associated with keloid formation. That may make it easier to understand why patients with pruritus have higher recurrence rate.

According to literature reports, the occurrence rates of radiotherapy related complications vary greatly and are related to multiple factors [29]. In our study, there were no patients with radiation induced dermatitis, bleeding, and poor wound healing. During treatment and follow-up, infection occurred in 2 patients and cutaneous fibroblastoma in 1 patient. The incidence of infection was significantly lower than that reported in the literature with 4.3-8% [9]. At present, there are few reports of cancer caused by radiation therapy for keloids. Biemans et al. reported a case of fibrosarcoma, which may be related to the carcinogenic effects of radiotherapy. In addition, Raghuvanci et al. reported 5 cases of keloid secondary malignancy after radiotherapy, but there is still no definite conclusion [13]. There were no patients developed cutaneous malignant in the radiotherapy field in this study. However, the cutaneous fibroblastoma was potentially associated with radiotherapy.

There are several limitations for this study. Firstly, the retrospective design of this study may result in bias that impact the accuracy of conclusion. Secondly, the single-center design makes it difficult to widely generalize the radiotherapy protocol. Therefore, a prospective, multicenter, randomized controlled trials are needed to confirm the result of this study.

## Conclusion

This study suggested that surgery followed by short-course every other day radiotherapy could achieve a satisfactory local control rate for keloid. Pain and or pruritus symptom was an independently prognostic factors for recurrence of keloid. A prospective randomized controlled study is need to further confirm this result.

### Electronic supplementary material

Below is the link to the electronic supplementary material.

Supplementary Material 1

Supplementary Material 2

Supplementary Material 3

## Data Availability

No datasets were generated or analysed during the current study.

## Abbreviations

BED: Biological effective dose
CI: Confidence intervals
HR: Hazard ratios
ROC: Receiver operating characteristic
RTOG: Radiation therapy oncology group
