# Supplementary material for: Treatment outcomes of surgery followed by short-course every other day radiotherapy in keloid
Source: Radiat Oncol. 2024 Jul 17;19:91. doi: 10.1186/s13014-024-02488-5 (PMC11256692; doi:10.1186/s13014-024-02488-5)
Supplement: Supplementary file 1 — Supplementary Material 1 [file 13014_2024_2488_MOESM1_ESM.docx]

Supplemental table 1. The 1 year. 3-year, 5-year local control rate estimates of with and without symptom.

| location | local control with pain and or pruritus (%) | | | | | | | local control without pain and or pruritus (%) | | | | | | | *p* | |  | |
| --- | --- | --- | --- | --- | --- | --- | --- | --- | --- | --- | --- | --- | --- | --- | --- | --- | --- | --- |
|  | 1year | | 3year | 5year |  | | | 1year | | 3year | | 5year |  | | |  | |  |
| ear | 60.0 | 26.7 | | 26.7 | |  | 97.9 | | 94.7 | | 94.3 | | |  | | ＜0.001 | |  |
| head neck | 36.4 | 36.4 | | 36.4 | |  | 97.0 | | 93.9 | | 93.9 | | |  | | ＜0.001 | |  |
| chest | 32.0 | 4.0 | | 4.0 | |  | 97.4 | | 97.4 | | 94.7 | | |  | | ＜0.001 | |  |
| abdomen | 50.0 | 25.0 | | 8.3 | |  | 95.0 | | 95.0 | | 95.0 | | |  | | ＜0.001 | |  |
| perineum | 54.5 | 45.5 | | 18.2 | |  | 96.0 | | 96.0 | | 96.0 | | |  | | ＜0.001 | |  |
| limb / back | 33.3 | 0.0 | | 0.0 | |  | 88.9 | | 88.9 | | 88.9 | | |  | | ＜0.001 | |  |
