# Supplementary figures and images for: Treatment outcomes of surgery followed by short-course every other day radiotherapy in keloid
Source: Radiat Oncol. 2024 Jul 17;19:91. doi: 10.1186/s13014-024-02488-5 (PMC11256692; doi:10.1186/s13014-024-02488-5)

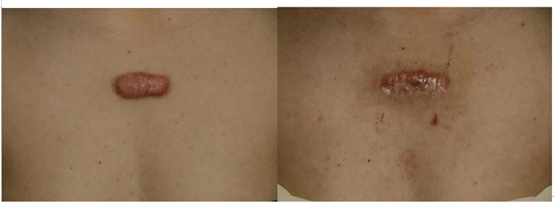

Supplement: Supplementary file 2 — Supplementary Material 2 [file 13014_2024_2488_MOESM2_ESM.png]

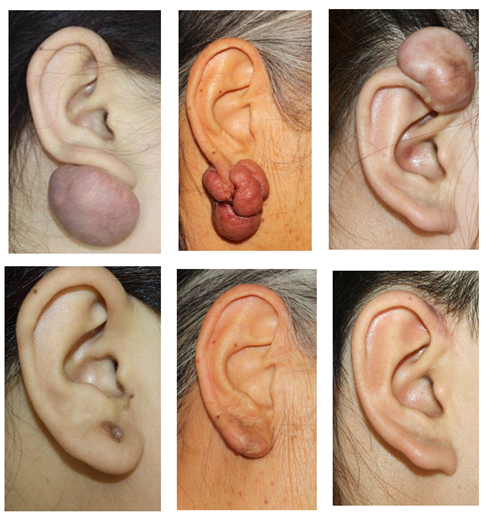

Supplement: Supplementary file 3 — Supplementary Material 3 [file 13014_2024_2488_MOESM3_ESM.png]
